# Supplementary material for: Eco-Friendly Bioemulsifier Production by Mucor circinelloides UCP0001 Isolated from Mangrove Sediments Using Renewable Substrates for Environmental Applications
Source: Biomolecules. 2020 Feb 27;10(3):365. doi: 10.3390/biom10030365 (PMC7175160; doi:10.3390/biom10030365)
Supplement: Supplementary file 1 [file biomolecules-10-00365-s001.pdf]

Supplementary Materials

Eco-Friendly Bioemulsifier Production by *Mucor circinelloides* UCP0001 Isolated from Mangrove Sediments Using Renewable Substrates for Environmental Applications

Nathália S.A.A. Marques [1,2,3], Israel G. Sales da Silva [1], Davi L. Cavalcanti [2,3], Patrícia C.S.V. Maia [1,2,3], Vanessa P. Santos [1,2,3], Rosileide F.S. Andrade [3] and Galba M. Campos-Takaki [3,*]

1 Northeast Biotechnology Network, Federal Rural University of Pernambuco, Recife-PE 52171-900, Brazil nathaliasa13@hotmail.com (N.S.A.A.M.); israelgsds@gmail.com (I.G.S.d.S.); patriciacvsm@gmail.com (P.C.S.V.M.); nessapimentel4519@hotmail.com (V.P.S.)

2 Doctorate Program in Biological Sciences, Federal University of Pernambuco, 50.870-420 Recife, Pernambuco, Brazil; davicavalcanti25@gmail.com

3 Nucleus of Research in Environmental Sciences and Biotechnology, Catholic University of Pernambuco, 50050-590, Recife, Pernambuco 50050-590, Brazil; rosileide_fontenele@yahoo.com.br

The factors that most influence the response variable is the quadratic effect of the corn steep liquor; the effect of the linear interaction between the waste cooking oil and the corn steep liquor; quadratic effect of residual frying oil and the linear effect of corn steep liquor. It is also remarkable that the lack of adjustment was not significant, which indicates that the proposed mathematical model fits well the observed and predicted data (**Figure S1)**.

The results showed the capacity of the emulsifier was retained under extreme variation in salinity concentrations (0–50 g NaCl L−1), pH (3–10) and different temperatures (25–121 °C), as well as pH indicated remarkable stability.

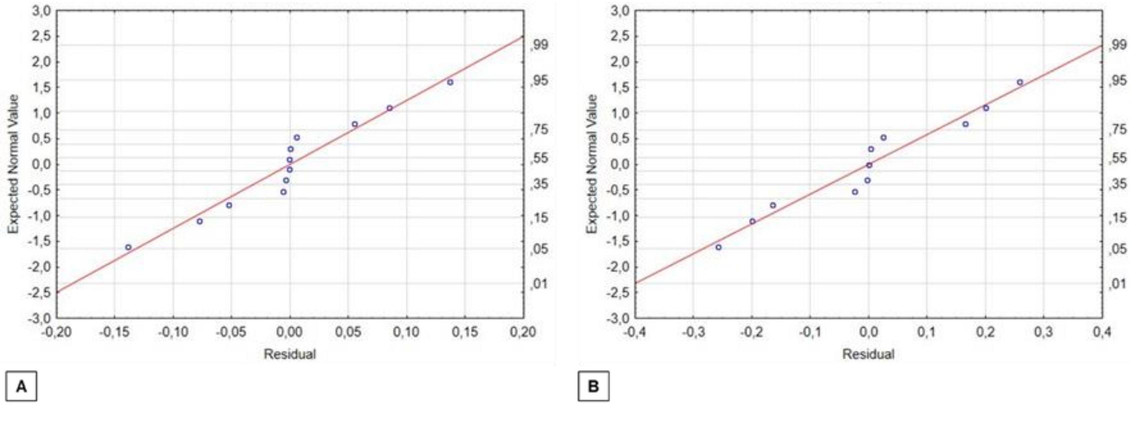

Figure S1. Normality chart of the residues. (A) EA in canola oil and (B) EA in burnt engine oil

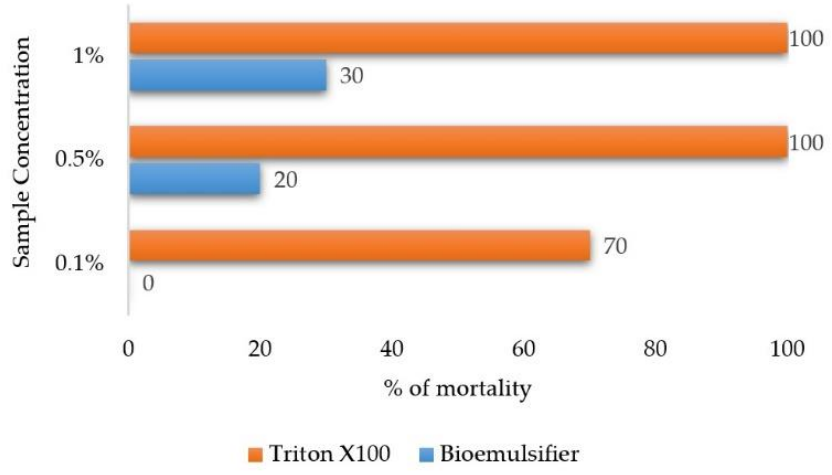

Figure S2. Toxicity test of the bioemulsifier using Artemia salina as a bioindicator.

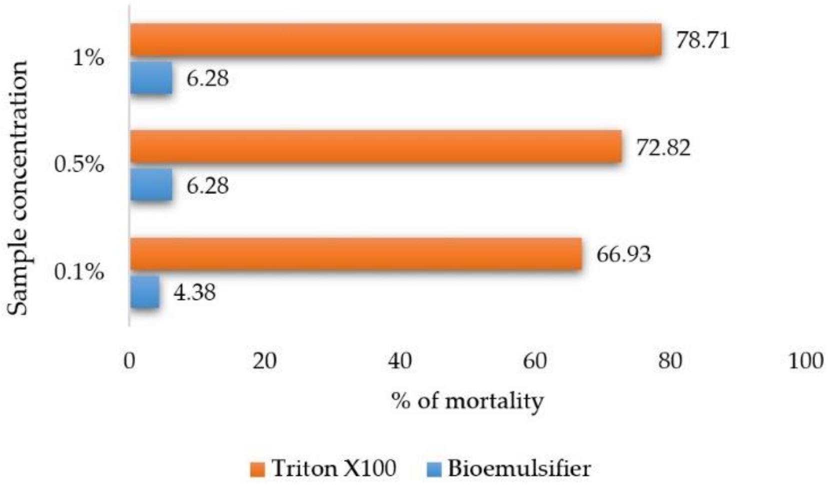

Figure S3. Phytotoxity test of the bioemulsifier using Chlorella vulgaris as a bioindicator.

These results demonstrate the hydrophobic tail composition and the molecule showed carbohydrate–protein–lipid complexes. The confirmation of carbohydrate, protein and fatty acid components in the isolated bioemulsifier confers upon them better emulsifying potential and ability to stabilize emulsions (Figure S4).

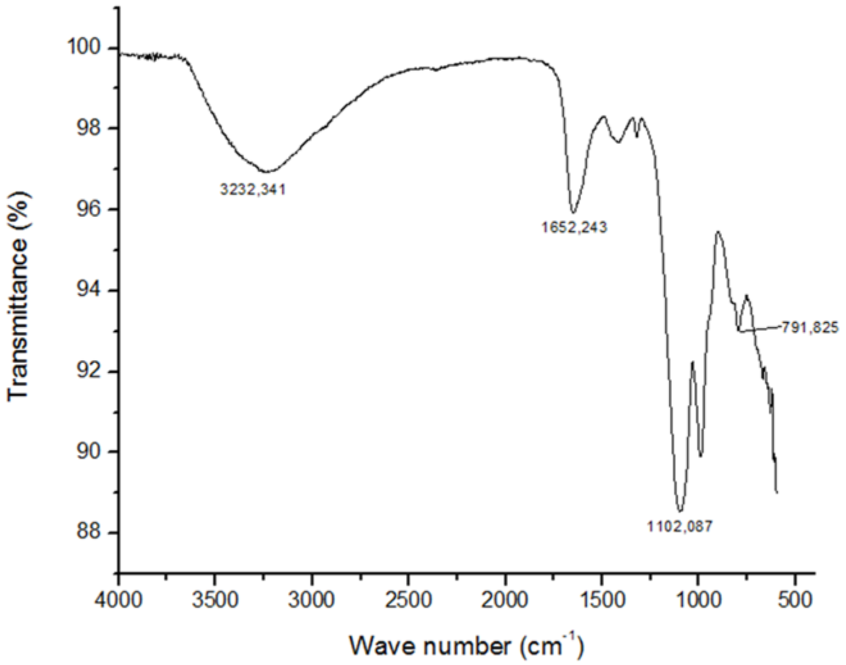

Figure S4. Fourier Transform Infrared (FT-IR) spectrum of *Mucor circinelloides* UCP0001 bioemulsifier
